# The Effect of a Leisure Time Physical Activity Intervention Delivered via a Workplace: 15-Month Follow-Up Study

**DOI:** 10.3390/ijerph15020264

**Published:** 2018-02-04

**Authors:** Marit Skogstad, Lars-Kristian Lunde, Bente Ulvestad, Hans Christian D. Aass, Thomas Clemm, Asgeir Mamen, Øivind Skare

**Affiliations:** 1 Department Occupational Medicine and Epidemiology, National Institute of Occupational Health, Box 8149 Deptartment, 0033 Oslo, Norway; Bente.Ulvestad@stami.no (B.U.); Oivind.Skare@stami.no (Ø.S.); 2 Department for Work Psychology and Physiology, National Institute of Occupational Health, Box 8149 0033 Oslo, Norway; Lars-Kristian.Lunde@stami.no; 3 The Blood Cell Research Group, Department of Medical Biochemistry, Oslo University Hospital, 0450 Ullevaal, Norway; h.c.aass@medisin.uio.no; 4 Occupational Health Service Department, Mesta AS, Fanaveien 221 C, 5239 Rådal, Norway; Thomas.Clemm@mesta.no; 5 Norwegian School of Health Sciences, Kristiania University College, Box 1190 Sentrum, 0107 Oslo, Norway; asgeir.mamen@nhck.no

**Keywords:** physical activity, cardiovascular, occupational health

## Abstract

In line with recommendations from both the World Health Organization and the European Union some employers encourage workplace health promotion through physical activity (PA) facilities and leisure time PA-initiatives. The current study describes a 15-month follow-up after an 8-week workplace delivered PA-initiative, investigates if improvements in cardiovascular risk status are sustainable, and elucidates differences according to educational level. One hundred and twenty one employees (43 women) were examined before and after the 8-week PA-initiative and 94 at the 15-month follow-up. PA-levels, blood pressure, resting heart rate, lipids, glycosylated hemoglobin (HbA1c), C-reactive protein (CRP), and selected markers of inflammation were registered at baseline, immediately after the 8-week PA-initiative, and 15 months after baseline. At the end of follow-up (15-month), PA-levels—increased during the 8-week intervention—had returned to baseline values. None of the five improvements in cardiovascular markers (total cholesterol, low density lipoprotein (LDL), P-selectin, CD40Ligand (CD40L) and Monocyte chemoattractant protein-1 (MCP-1)) seen at the 8-week follow-up were sustained. At the 15-month follow-up as compared to baseline HbA1c, CRP (log) and interleukin-6 (IL-6) were reduced by 0.06 mmol/L (95% CI −0.11,−0.01), 0.25 mg/L (95% CI −0.46,−0.04) and 0.39 pg/mL (95% CI −0.75, −0.04), respectively. At baseline, there were differences in cardiovascular risk factors comparing men with low versus high levels of education. No differences in changes in outcomes between these groups of men were found during follow-up. In this study highly educated men generally have lower levels of cardiovascular risk factors, but the effect of PA on such markers in this group do not differ from the effects seen in less educated men.

## 1. Introduction

Studies during the last 60 years have shown associations between physical activity (PA) and lower risk of mortality due to cardiovascular disease and cancers [1,2,3,4,5]. PA may have favorable physiological effects on blood pressure, blood lipids [6,7], glycemic control [8] and systemic inflammation [9]. There are however few studies exploring the effects of workplace-initiated leisure-time PA on physical health parameters. A population-based study showed that adherence to health behavior recommendations was low concerning PA and non-sedentary behavior among middle-aged and older adults [10]. Interventions in working life may be more advantageous. A 1-year follow-up study among Danish cleaners showed an aerobic exercise adherence of 64% after 4 months [11] whereas another Scandinavian study reported adherence of 70% after 30 months [12]. Systematic reviews have found that most publications in this field used individual counseling as the intervention [13,14,15,16]. Further, the outcomes in these studies are often limited to self-reported change in PA or BMI, and “meaningful outcomes” like blood pressure and blood lipid level are mostly lacking [13,15,16,17]. A review by Groeneveld et al. [14] found no evidence for an effect of intervention on serum lipids, blood glucose nor blood pressure. Cohort studies have mostly been carried out with a limited number of participants, few blue-collar workers and response rates have been low [13]. Follow-up periods have mostly been short, lasting only weeks or a few months [13,14,17,18]. These reviews conclude that the results from included studies are inconclusive as to whether workplace PA-interventions are efficacious [15,18], but that interventions evaluated in high quality studies may influence PA-behavior [16]. 

Some employers have made PA facilities available at the workplace and have initiated PA-initiatives for leisure time. This is in line with recommendations from both the World Health Organization and the European Union who encourage workplace health promotion [19]. 

The current study describes the 15-month follow-up from a cohort of office and road workers that took place in an eight-week leisure time PA-program initiated by the employer [20,21]. The enterprise management organized the 8-week unsupervised intervention in which the idea was to increase PA. Here, the participants competed against each other individually and in self-selected teams in a virtual internet mountain track. Cardiovascular risk factors were investigated before and after the eight weeks of leisure time PA. Beneficial effects on lipid status and an increase in maximal oxygen uptake (VO_2max_) were found at the end of the intervention [20]. The markers of inflammation P-selectin and CD40 L were decreased in the total group, along with a decrease in MCP-1 among men [21]. The study included both women and men. The women were all, with an exception of two individuals, highly educated office workers. Since the group of men consisted of one group of office workers and another one of road workers, cardiovascular risk factors between men with low and high levels of education were aimed to be elucidated. Educational level might be an important factor in the selection into PA-programs and to the sustainability of these programs. Workers in manual occupations run a higher risk of cardiovascular disease compared to generally higher educated office workers [22,23]. This increased risk in manual workers does not apply to those who exercise [22,23]. 

The main aim of the present study was to assess if the improvements in cardiovascular risk status following the PA-intervention were sustainable 15 months after baseline registration and to see if the changes in outcome differed between men with a low versus a high level of education. 

## 2. Materials and Methods 

### 2.1. Study Setting and Design

**Figure 1:**
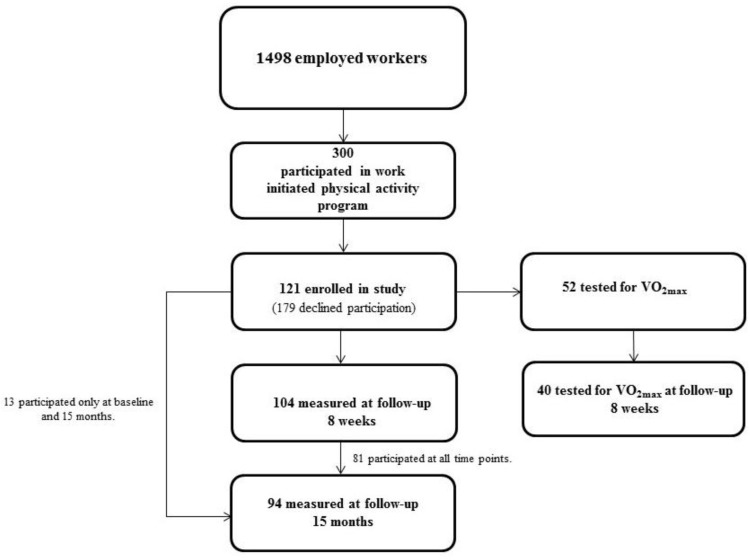
Flow diagram for the study.

A detailed description of the cohort has been presented elsewhere [20,21]. Briefly, a subgroup of 121 individuals from a road maintenance company with altogether 1498 workers throughout Norway, were enrolled in the study (Figure 1). The initial cohort consisted of 78 men (64%) and 43 women, with a mean age of 41.8 years (SD 12) and 42.6 years (SD 12.5), respectively. Among these participants, 86% of the women and 53% of the men reported college or university education. All but two women were office workers, while 29 men (24% of the total group) were road workers (see Table 1). The examinations took place before the start of eight weeks of PA in September 2014 (baseline), after the PA-period (8-week follow-up) in November/December 2014 and then in November/December 2015 (15-month follow-up). The Regional Ethics Committee in Oslo (2014/1521) approved of the study. All participants were informed about the study and gave written consent to participate.

Based on the self-determination theory which may be favorably applied to health contexts [24,25], and believing that behavioral changes occur through group dynamic processes, the enterprise management organized an 8-week activity intervention, running from September to November 2014. The intention of the PA-program was to motivate employees to increase leisure PA. The participants competed against each other individually and in teams (2–8 members) in a virtual internet mountain track. Participants moved forward by registering daily steps with a wristband pedometer (Tappa^®^, IDT, Hong Kong, China). The PA activities were home-based, self-selected (amount of aerobic exercise and resistance training was by own choice), and performed unsupervised. Participants had online access to individual and team’s progress, in addition to best daily performers and overall leaders. Activities not measured by the pedometer (e.g., resistance training, swimming, bicycling, and spinning), could be converted into steps and registered. The best individual performer and team received a prize at the end of the PA-period. Information about the activity program was spread throughout the company via information meetings, the enterprise newspaper and the enterprise intranet for two months prior to start up. All employees were invited to participate [20,21]. 

VO_2max_ was measured at baseline and after the 8-week PA-initiative and tested using a cycle ergometer (Monark 874E, Monark Exercise AB, Vansbro, Sweden). Here, the resistance was increased every minute by 35 W until the subject was exhausted (cadence < 65 RPM), oxygen uptake was measured continuously and VO_2max_ was calculated from the highest 30 s averaging interval at the conclusion of the test [20,21]. Due to technical and practical reasons, the VO_2max_ test was not included at final follow-up (15 months). Instead, self-reported weekly low/moderate (e.g., walking, gardening, playing golf) and high intensity PA (e.g., running, swimming, cycling with moderate/high pulse) was registered at the 15-month follow-up. 

Number of days per week of PA was collected by questionnaire on all three occasions and assessed by the question: How often do you normally exercise, with the response alternatives: never, less than once per week, once per week, two to three times per week or 4 or more times per week [26]. Since the distribution of reported PA was skewed, PA was arranged into three activity groups: once a week or less, 2–3 times per week, and 4 or more times per week. 

Three months prior to the 15-month follow-up, the employer offered a new PA-initiative of 8 weeks duration. Forty-seven participated, but this PA-initiative was not part of the present study. 

Blood pressure and RHR were measured three times with BpTRU^®^ (Bp TRU Medical Devices, Coquitlam, BC, Canada). The measurements were taken on all three occasions and the procedure has been described elsewhere [20,21]. 

### 2.2. Blood Analyses

Non-fasting blood (tubes containing K_2_EDTA or serum Sep Clot Activator) was sampled at the same time of the day on all three occasions for investigation of lipids (cholesterol, low-density lipoprotein (LDL), high-density lipoprotein (HDL)), glycosylated hemoglobin (HbA1c), C-reactive protein (CRP) and HbA1c. The serum tubes were centrifuged at the workplace of the participants and the blood samples were delivered to the Department of Medical Biochemistry, Oslo University Hospital who analyzed the samples within 24 h.

Cholesterol, LDL and HDL in serum were analyzed by an enzymatic colorimetric method in the Cobas 8000 c702. HbA1c in EDTA blood was analyzed with a D100 TM system (Bio-Rad, Hercules, CA, USA) which uses “high performance liquid chromatography” as the separation principle, whereas CRP in serum was quantified by particle enhanced immunoturbidimetric method with a Cobas 8000 instrument (Cobas 8000 c702, Roche Diagnostics, Tokyo, Japan). 

Blood for cytokines was collected in S-Monovette 7.5 mL Serum geltubes (Sarstedt AG & Co., Nümbrecht, Germany, Cat. No. 1602) and left for 30 to 60 min at room temperature prior to centrifugation for 15 min. Serum was collected, mixed and aliquoted into approximately 500 μL volumes in 1.8 mL Nunc tubes and stored at −80 °C and analyzed according to standards at the University Hospital, details are provided in former publications [20,21]. 

### 2.3. Statistical Analysis 

Baseline sex differences were analyzed by linear regression adjusting for age and education. Differences between men with a high level of education and those with a low level of education were tested similarly, without adjustment for education. Linear mixed models were applied to study the association between follow-up and each of the outcome variables (systolic blood pressure (sBP), diastolic blood pressure (dBP), RHR, total cholesterol, LDL, HDL, HbA1c, CRP, IL-6, MCP-1, TNF-α, P-selectin, CD40L, leptin, adiponectin) collected at baseline and the 8-week and 15-month follow-ups. The CRP and leptin values were ln-transformed prior to analysis to give a better fit to the normal distribution. The mixed model analyses were adjusted for age, sex, smoking, baseline PA, participation in a second PA-initiative and level of education. The mixed models were applied to the whole sample and stratified according to sex. Further, by adding an interaction between follow-up and education, it was investigated whether the adjusted change in outcome from baseline to follow-up differed between educational groups. For the analysis of sBP, dBP, RHR, total cholesterol, LDL, HDL, HbA1c and CRP, a random intercept was added for subject. For the other variables, kit and plate variation was taken into account by including for random intercepts for kit and plate, with plate nested in kit, which were crossed with the random intercept of subject. All analyses were done in R (htttp://www.r-project.org).

## 3. Results

Among the 121 examined at baseline, 104 and 94 participated at the 8-week and 15-month follow-up, respectively. Compared to the ones who remained in the cohort throughout the study period, the dropouts at the two follow-ups were less educated, had lower HDL, and higher CD40L (results not shown) [20,21]. 

**Figure 2:**
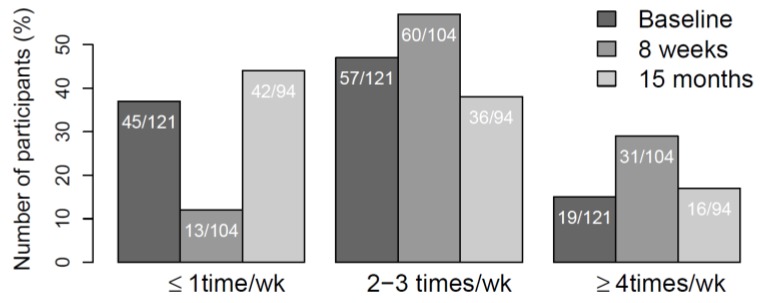
Change in weekly physical activity level; baseline, the 8-week and 15-month follow-up.

After the initial increase seen at the 8-week follow-up, levels of PA returned to baseline values (Figure 2). At the final follow-up (15-month), 60 participants reported to have the same activity level as compared to baseline values, 16 reported more, and 18 less activity. Compared to the 8-week follow-up, 43 participants had the same activity level, six had more and 32 had less activity. At the final follow-up (15-month), the group reported a mean weekly low/moderate and high PA of 240 min (SD = 257) and 123 (SD = 135), respectively. 

**Figure 3:**
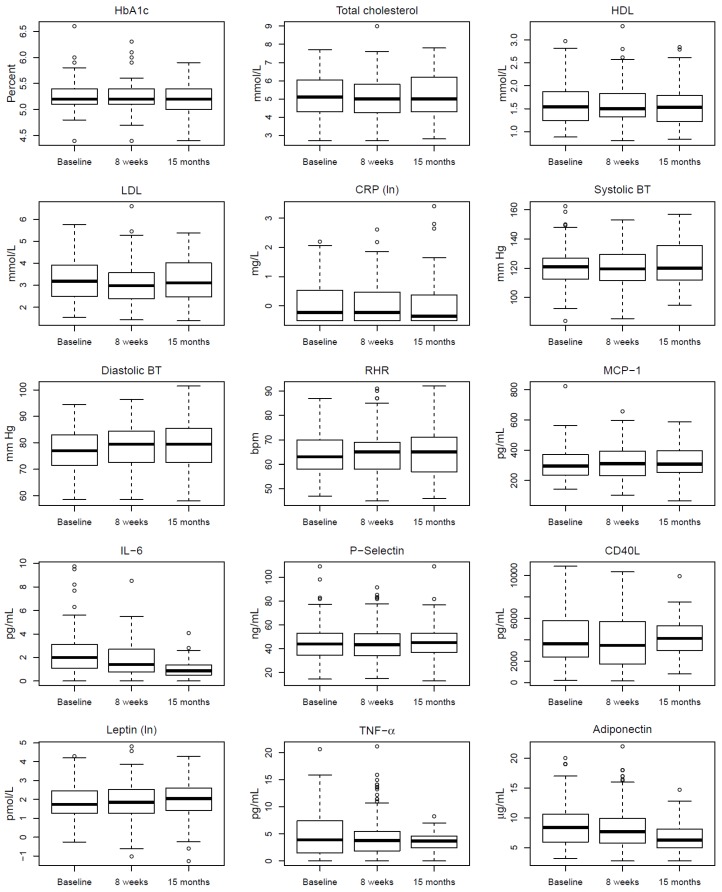
Changes in HbA1c, lipids, CRP, Blood pressure, RHR and selected biomarkers according to time; baseline, 8-week follow-up and 15-month follow-up. Plots are based on participants observed at all three time points.

*Diastolic and systolic blood pressure* (*dBP*/*sBP*). The significant increase in dBP registered at the 8-week follow-up [20] did not persist in the period from 8 weeks to 15 months. For the period from baseline to 15 months of follow-up, there was, however, a significant increase in dBP and sBP for men. See Table 2, Figure 3. 

*Resting heart rate* (*RHR*). The RHR did not change during the 8-week PA period [20]. However, there was a significant increase among women during the follow-up of 15 months. See Table 2, Figure 3. 

*Total cholesterol*. Total cholesterol decreased during the PA-initiative of eight weeks [20] but increased significantly between the 8-week and 15 months of follow-up. See Table 3, Figure 3. 

*Low Density Lipoprotein*. LDL also showed a reduction at the 8-week follow-up [20], but increased between the end of the PA-period and 15 months of follow-up. See Table 3, Figure 3. 

*High density Lipoprotein*. HDL did not change during the 8-week PA initiative [20] nor during the 15-month follow-up. See Table 3, Figure 3. 

*Glycosylated hemoglobin* did not change during the 8-week PA initiative, but was significantly reduced at the 15-month follow-up. This was compared to the baseline of 0.06 mmol/L, 95% CI −0.11,−0.01, *p* = 0.014. See Table 3, Figure 3.

*CRP* and *IL-6* did not change during the eight weeks of PA-initiative [20,21] but were reduced at the 15-month follow-up when compared to baseline values of 0.25 mg/L (95% CI −0.46, −0.04, *p* = 0.019) and 0.39 pg/mL (95% CI −0.75, −0.04, *p* = 0.032), respectively. See Table 4, Figure 3.

*MCP-1* decreased among men during the 8-week PA period [21] but no change was found between baseline and 15 months of follow-up. For women, it increased significantly from baseline to the 15-month follow-up. See Table 4, Figure 3. 

TNF-α did not change during the 8-week of PA [21], nor during follow-up. See Table 4, Figure 3. 

P-selectin and CD40L were reduced during the 8-week PA period [21], but increased significantly in the following period. Compared to baseline, P-selectin returned to start-up levels, and CD40L was significantly increased, See Table 5, Figure 3.

*Leptin* and *ddiponectin* did not change during the eight weeks of PA [21] and remained unchanged for the entire follow-up, See Table 5, Figure 3.

*Educational level*. At baseline, the number of cardiovascular risk factors of the men with a low level of education were more pronounced than what was the case for the men with a high level of education, See Table 1. There was no clear difference in change during follow-up of these cardiovascular risk factors when these two groups of men were compared to each other (results not shown).

## 4. Discussion 

The improvement in cardiovascular risk status achieved during an 8-week PA-initiative [20,21], was not sustainable at the 15-month follow-up. However, HbA1c and the two markers of inflammation; CRP and IL-6 were reduced at the 15-month follow-up. 

Supervised high intensity PA increases cardiorespiratory fitness in an occupational setting [27]. Such fitness achieved through high intensity PA such as running, bicycling and swimming reduces the stiffness of the arteries through vasodilatory NO-release [28] and diminishes oxidative stress and inflammation [29]. Even though PA returned to baseline values at the final follow-up in the present study, the mean weekly moderate and high PA (MVPA) exceeded the national recommendations of 150 min per week [30]. VO_2max_, increased during the 8-week follow-up [20,21]. In accordance with exercise interventions [31], more beneficial lipid figures were found among those who reported regular high weekly PA compared with those who reported PA once or less each week in the present cohort [20]. Furthermore, a decrease in HbA1c at the 15-month follow-up compared to baseline values was found. HbA1c reflects values even 120 days prior to blood sampling which took place in the winter, so the results obtained could reflect values in the fall when the participants could be more physical active due to weather conditions. PA has a positive effect on the insulin sensitivity [2]. A reduction of CRP and IL-6 by PA is also beneficial since there is an increased vascular risk with high levels of these biomarkers [32,33] and even some cancers [34]. Regular PA reduces resting CRP levels by a decrease in cytokine production in adipose tissue, by indirectly improving endothelial function and increasing insulin sensitivity and by inducing an antioxidant effect [35]. Compared to baseline values, a reduction in CRP and IL-6 was found at the final follow-up. All in all these results indicate that PA-interventions at the workplace may promote favorable health effects, among others preventing cardiorespiratory diseases and even some cancers. 

Women in the cohort consisted mostly of participants with a high degree of education employed as office workers. Compared to the men these women had less cardiovascular risk factors e.g., lower blood pressure, higher levels of HDL and adiponectin, and lower biomarkers of inflammation such as P-selectin, MCP-1 and TNF-α. These differences could reflect sex differences. However, during the 15-month follow-up there were few significant changes of these parameters among the women. At the baseline registration, the men were quite similar to each other regarding anthropometrics. Still, the men with a low level of education had a tendency of more cardiovascular risk factors, such as higher RHR and inflammation (TNF-α and IL-6), along with having significantly higher HbA1c, CD40L, Leptin and lower HDL. Men with a low level of education were also less physically active and had lower VO_2max_ compared to the higher educated men [20,21]. The men with a low level of education worked long work-shifts and had mostly sedentary work and most of the working day was spent in construction lorries. Long work-shifts combined with sedentary work is of concern since studies of manual workers show increased mortality and a higher risk of cardiovascular disease, but not among those who exercise during leisure time [22,36]. During the eight-week motivational PA intervention, the men with a low level of education increased VO_2max_ [20]. Overall, the changes obtained during the 8-week of PA were not sustainable during the last follow-up period, similar to what was the case among the men with a high level of education. 

The present study demonstrates a high participation frequency of 86% and 78% at first and last follow-up, respectively. This, along with a rather high number of participants [29,37] and its prospective design, are other strengths of the study. Also, compared to more clinically based studies with participants with cardiovascular disease, metabolic syndrome or even cancer patients [29,37], a high number of cardiovascular risk markers in a healthy population was used in the present study. 

Mixed models provide an adequate method for analyzing longitudinal data. These models are robust to dropout that is missing at random, flexible in the choice of variance structures, and utilize all available observations including those from participants with non-complete observations.

A limitation of the study is that exercise levels were self-reported. Furthermore, there was a self-selection of participants into the study so that healthy, younger, highly educated and possibly motivated individuals were overrepresented. Thus, at baseline the cohort included fewer blue-collar workers (24%) as compared to the entire company (74%) [20]. 

It is plausible that a stronger intervention-outcome is possible in a controlled laboratory setting. However, compared to the current study, such studies mostly have less participants, measure fewer cardiovascular risk factors and study other populations. Therefore, the presented study may give a better picture of how a PA-intervention can affect health outcomes in occupational settings. 

## 5. Conclusions 

Participants reported an increase in leisure-time PA during the 8-week follow-up, the PA was back to baseline levels at the final follow-up (15-month). The initial improvements in cardiovascular risk factors were generally not sustainable at the 15-month follow-up. Initially, the men with a low level of education had less favorable health than those with a high level of education. Furthermore, there were no clear difference between those with a low and high level of education with regard to the sustainability of the health parameters. Single PA-interventions have limited effect on long-term health outcome. Based on the findings of the present study, PA-interventions initiated by the employer may need to be repeated on a regular basis for improvements to be sustainable. 

## Figures and Tables

**Table 1:** Characteristics at baseline among females and males participating in the study.

Outcome	Men Low Education (N = 37) ^a^ Mean (SD)	Men High Education (N = 41) ^e^ Mean (SD)	Women All (N = 43) ^b^ Mean (SD)
Age (years)	43.0 (12.9)	40.8 (11.1)	42.6 (12.5)
BMI (kg/m^2^)	27.3 (4.8)	25.4 (3.0) ^†^	24.4 (3.1)
RHR (bpm) Smokers ^c^	65.7 (9.9) 6	61.3 (8.5) ^†^ 6	67.4 (10.9) * 0 *
Systolic BP (mmHg)	123.3 (13.2)	122.3 (11.5)	112 (16) *
Diastolic BP (mmHg)	78.5 (9.5)	79.2 (87.8)	74 (8) *
CRP (mg/L)	2.3 (2.3)	1.5 (1.7)	1.9 (1.8)
Cholesterol (mmol/L)	5.0 (1.1)	5.1 (1.1)	5.4 (1.1)
HDL (mmol/L)	1.3 (0.3)	1.4 (0.3) ^†^	1.9 (0.4) *
LDL (mmol/L)	3.2 (0.9)	3.3 (1.0)	3.2 (0.9)
HbA1c (mmol/L)	5.4 (0.4)	5.2 (0.3) ^†^	5.2 (0.3)
VO_2max_^d^ (mL/kg min)	35.8 (5.3)	40.8 (6.7)	34.1 (9.5) *
IL-6 (pg/mL)	2.9 (2.1)	2.1 (1.8)	2.1 (1.4)
MCP-1 (pg/mL)	317.0 (120.7)	332.6 (126.3)	288.3 (89.8) *
TNF-α (pg/mL)	7.5 (4.3)	5.1 (3.6) ^†^	3.1 (3.1) *
P-selectin (ng/mL)	50.9 (18.7)	45.9 (16.8)	41.0 (10.6) *
CD40L (pg/mL)	4864 (2756)	3336 (2134) ^†^	4742 (2590) *
Leptin (µg/mL)	8.7 (7.6)	5.3 (4.4) ^†^	16.0 (13.6) *
Adiponectin (µg/mL)	6.6 (3.2)	7.5 (2.6)	10.5 (3.8) *

Significantly different between sex * *p* < 0.05, ^a^ N = 35 for CRP, N = 36 for HbA1c. ^b^ N = 42 for BMI and HbA1c, N = 41 for LDL, HDL and cholesterol. ^c^ Total number. ^d^ N = 12 for men with low education, N = 20 for men with high education, N = 20 for women. ^e^ N = 40 for CRP, HDL, LDL and cholesterol, 38 for HbA1c. ^†^ Significantly different between the two groups of men.

**Table 2:** Systolic blood pressure, diastolic blood pressure, and resting heart rate.

			Females N = 43				Males N = 78				All N = 121			
Outcome	Unit	Time	β	95% CI		*p*	β	95% CI		*p*	β	95% CI		*p*
Syst. BP	(mmhg)	15 months vs. BL	−0.81	−5.88	4.27	0.752	3.92	0.42	7.42	0.029	2.27	−0.59	5.13	0.119
		15 months vs. 8 weeks	0.57	−4.55	5.69	0.824	2.44	−1.20	6.07	0.187	1.82	−1.12	4.76	0.223
Diast. BP	(mmhg)	15 months vs. BL	−0.22	−3.77	3.34	0.904	2.99	0.61	5.37	0.014	1.86	−0.11	3.83	0.064
		15 months vs. 8 weeks	−0.56	−4.15	3.03	0.756	0.59	−1.89	3.06	0.640	0.19	−1.83	2.21	0.851
RHR	(bmp)	15 months vs. BL	4.03	0.49	7.58	0.026	0.17	−2.64	2.99	0.903	1.18	−1.07	3.43	0.303
		15 months vs. 8 weeks	5.04	1.47	8.62	0.006	−1.24	−4.16	1.68	0.402	0.70	−1.61	3.01	0.553

**Table 3:** Cholesterol, LDL, HDL, and HbA1c.

			Females N = 42				Males N = 77				All N = 119			
Outcome	Unit	Time	β	95% CI		*p*	β	95% CI		*p*	β	95% CI		*p*
Cholest.	(mmol/L)	15 months vs. BL	−0.04	−0.31	0.22	0.741	0.11	−0.08	0.31	0.256	0.05	−0.10	0.21	0.500
		15 months vs. 8 weeks	0.25	−0.02	0.52	0.072	0.11	−0.09	0.32	0.270	0.17	0.01	0.33	0.043
LDL	(mmol/L)	15 months vs. BL	−0.05	−0.24	0.15	0.645	0.10	−0.06	0.27	0.226	0.05	−0.08	0.18	0.440
		15 months vs. 8 weeks	0.23	0.03	0.43	0.024	0.14	−0.03	0.32	0.114	0.18	0.05	0.31	0.009
HDL	(mmol/L)	15 months vs. BL	0.01	−0.09	0.11	0.856	−0.04	−0.09	0.01	0.151	−0.02	−0.07	0.02	0.330
		15 months vs. 8 weeks	0.06	−0.04	0.15	0.266	−0.04	−0.09	0.01	0.134	−0.01	−0.06	0.04	0.750
HbA1c	(mmol/L)	15 months vs. BL	−0.05	−0.14	0.04	0.301	−0.06	−0.11	−0.01	0.028	−0.06	−0.11	−0.01	0.014
		15 months vs. 8 weeks	−0.02	−0.12	0.07	0.617	−0.05	−0.10	0.01	0.080	−0.04	−0.09	0.01	0.088

**Table 4:** CRP, IL-6, MCP-1 and TNF-α.

			Females N = 42				Males N = 77 ^a^				All N = 119 ^b^			
Outcome	Unit	Time	β	95% CI		*p*	β	95% CI		*p*	β	95% CI		*p*
logCRP	(mg/L)	15 months vs. BL	−0.18	−0.56	0.19	0.338	−0.28	−0.53	−0.03	0.030	−0.25	−0.46	−0.04	0.019
		15 months vs. 8 weeks	−0.06	−0.44	0.32	0.737	−0.16	−0.42	0.10	0.234	−0.13	−0.34	0.08	0.232
IL-6	(pg/mL)	15 months vs. BL	0.00	−0.51	0.52	0.994	−0.62	−1.1	−0.13	0.013	−0.39	−0.75	−0.04	0.032
		15 months vs. 8 weeks	0.14	−0.34	0.62	0.572	−0.34	−0.8	0.12	0.154	−0.18	−0.51	0.16	0.310
MCP-1	(pg/mL)	15 months vs. BL	41.36	7.10	75.61	0.020	6.82	−24.40	38.04	0.669	18.63	−5.23	42.50	0.127
		15 months vs. 8 weeks	20.75	−11.69	53.20	0.212	19.31	−10.86	49.48	0.211	18.80	−4.02	41.61	0.107
TNF-α	(pg/mL)	15 months vs. BL	0.17	−0.94	1.28	0.761	−0.31	−1.27	0.65	0.523	−0.15	−0.93	0.62	0.697
		15 months vs. 8 weeks	−0.06	−1.11	0.99	0.907	0.01	−0.91	0.93	0.983	−0.06	−0.80	0.68	0.877

^a^ N = 75 for CRP, IL-6, TNF-α. ^b^ N = 117 for CRP, IL-6, TNF-α.

**Table 5:** P-selectin, CD40L, leptin, and adiponectin.

			Females N = 42				Males N = 77				All N = 119			
Outcome	Unit	Time	β	95% CI		*p*	β	95% CI		*p*	Β	95% CI		*p*
P-selec.	(ng/mL)	15 months vs. BL	1.55	−2.94	6.04	0.501	1.71	−2.41	5.83	0.417	1.75	−1.31	4.82	0.263
		15 months vs. 8 weeks	2.73	−1.53	6.98	0.211	3.30	−0.67	7.28	0.105	3.06	0.13	6.00	0.041
CD40L	(pg/mL)	15 months vs. BL	267.82	−903.72	1439.35	0.655	1149.37	316.46	1982.28	0.007	851.70	169.57	1533.83	0.015
		15 months vs. 8 weeks	803.57	−299.04	1906.19	0.155	1418.38	611.60	2225.16	0.001	1204.99	551.66	1858.32	0.000
Leptin ln	(µg/mL)	15 months vs. BL	0.10	−0.17	0.37	0.482	0.08	−0.11	0.28	0.408	0.09	−0.08	0.25	0.311
		15 months vs. 8 weeks	0.04	−0.22	0.31	0.744	0.10	−0.09	0.29	0.308	0.07	−0.09	0.23	0.369
Adipon.	(µg/mL)	15 months vs. BL	−0.45	−1.34	0.44	0.324	−0.01	−0.67	0.66	0.985	−0.20	−0.73	0.33	0.457
		15 months vs. 8 weeks	−0.49	−1.33	0.35	0.252	−0.11	−0.75	0.53	0.727	−0.28	−0.78	0.23	0.283
